# Effect of Die Geometry on the Formability of 5052 Aluminum Alloy in Electromagnetic Impaction Deformation

**DOI:** 10.3390/ma11081379

**Published:** 2018-08-08

**Authors:** Fei Feng, Jianjun Li, Rongchuang Chen, Peng Yuan, Hongliang Su, Qixian Zhang, Pan Huang, Zhizhen Zheng

**Affiliations:** State Key Laboratory of Materials Processing and Die & Mould Technology, School of Materials Science and Engineering, Huazhong University of Science and Technology, Wuhan 430074, China; fengfei@hust.edu.cn (F.F.); crc@hust.edu.cn (R.C.); yuanpeng@hust.edu.cn (P.Y.); sue@hust.edu.cn (H.S.); zhangqixian@163.com (Q.Z.); hphust@hust.edu.cn (P.H.); zzz@hust.edu.cn (Z.Z.)

**Keywords:** forming limit, high speed, strain rate, impaction, electromagnetic forming

## Abstract

The formability of aluminum alloy sheet in electromagnetic impaction deformation has attracted the attention of numerous researchers for the past decades. However, the influences of die geometry and high-speed impaction electromagnetic deformation on formability have not been well established, thereby resulting in the formability of the sheet not being developed fully. In this study, the influence of die geometry on the formability of 5052 aluminum alloy in electromagnetic deformation was investigated by comparing the formability of 5052 aluminum alloys formed using a hemispherical die and a cylindrical die. The intriguing finding is that the formability of the 5052 aluminum alloy formed using a cylindrical die is considerably higher than that formed using a hemispherical die. Therefore, die geometry significantly influences the formability of 5052 aluminum alloy. The influence of die geometry on the formability of 5052 aluminum alloy in high-speed impaction electromagnetic deformation was explained in terms of strain rate, pressure stress, and stress state. This investigation enhances insight into the interaction between sheets and dies, and provides a reference for the studying influence of dies on the forming limit of sheets in high-speed impaction deformation.

## 1. Introduction

Aluminum alloys have been investigated to verify their high potential for the aerospace field to improve fuel economy and reduce environmental pollution [1]. However, in the conventional forming processes, the forming limits of aluminum alloys are usually low. In general, the formability of aluminum alloy sheets can be increased during high strain rate deformation [2], such as during electromagnetic, electrohydraulic, and explosive forming processes. Electromagnetic forming technology exhibits an evident advantage over the other two methods because no forming medium is required, thereby making the electromagnetic forming more flexible and environment friendly. 

Many researchers have established that the formability of aluminum alloys can be improved to a certain extent via electromagnetic free forming. Tamhane et al. [3] investigated the electromagnetic expansion ring process of AA6061-T4 and pure copper, and reported a formability increase of approximately twice that of quasi-static ductility. The forming limits of Ti-6Al-4V and AA5052-O under quasi-static and electromagnetic forming processes were compared by Li et al. [4], and increases in formability by 24.37% and 10.97% were observed under electromagnetic forming conditions. Many scholars found that a higher formability increase can be achieved via electromagnetic die forming than via electromagnetic free forming because of the high-speed impact between the sheet and the die. Rohatgi et al. [5] found that the formability of 5182 aluminum could be locally increased by 2.6 times through electrohydraulic die forming than through electrohydraulic free forming. Imbert et al. [6] reported that the formability of 5754 aluminum alloy was evidently higher during electromagnetic die forming than during electromagnetic free forming, and the increase in formability was attributed to the interaction between the sheet and the die. Balanethiram et al. [7] attributed the formability increase in electromagnetic die forming to the high-velocity die-strike.

The manufacturing of an aluminum alloy thin-walled structure part such as a hemispherical shape workpiece used as a rocket storage tank has become a challenge due to the low forming limit of aluminum alloy. The formability of aluminum alloy is an intrinsic property which could also be affected by external process conditions, for example, the friction, strain rate, temperature, and the die geometry [8,9]. In the process of electromagnetic forming, the effect of discharge energy on the forming limit had been reported in our previous published paper [10,11]. The effect of strain rate and high-speed impaction on the forming limit had been investigated by Mala Seth et al. [9], they also specifically reported that sheet formability might depend largely on the die geometry in sheet high-speed impaction. However, the effect of die geometry on formability increase was not well investigated.

In this paper, an interesting phenomenon was observed in that the high-speed impaction was not always beneficial for the increase in forming limit. The boundary conditions (die geometry) would affect the formability of 5052 aluminum alloy sheet in high-speed impaction. Therefore, this article focuses on reporting the occurrence and the explanation of this phenomenon. The reasons for this phenomenon will be explained in terms of strain rate, pressure stress, and stress state. This investigation provides a deeper insight into the influence of boundary conditions on formability in high-speed impaction electromagnetic deformation. It will provide theoretical guidance to further increase the forming limit of aluminum alloys high-speed impaction electromagnetic forming.

## 2. Material and Experiment Procedures

### 2.1. Material

The as-received 5052 aluminum alloy sheet with a thickness of 1.0 mm was obtained via the tandem rolling. The chemical composition of the 5052 aluminum alloy sheet is listed in Table 1. 

### 2.2. Electromagnetic Impaction Forming Experiments

**Figure 1:**
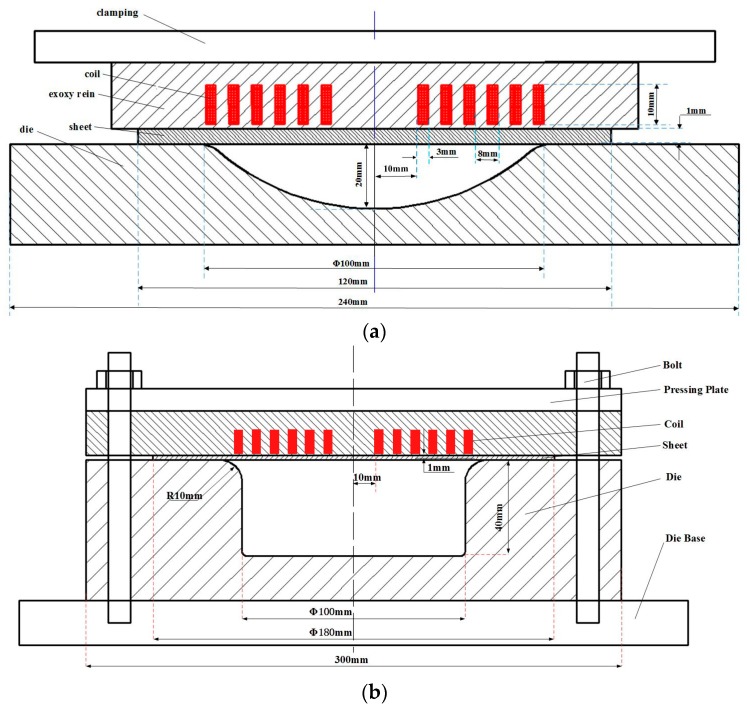
Schematic of the experiment devices (**a**) high-speed impaction electromagnetic deformation using a hemispherical die and (**b**) using a cylindrical die.

Figure 1 shows the schematic of the high-speed impaction electromagnetic deformation experiment, in which a hemispherical die and a cylindrical die were used. The high-speed impaction electromagnetic deformation using a hemispherical die is shown in Figure 1a. The 5052 aluminum alloy sheet was placed below a flat spiral coil, and then the coil was pressed by the hydraulic equipment to restrain the coil and sheet sliding. The dimensions of the coil and hemispherical die are also shown in Figure 1a. When the magnetic force reached the yield limit of the aluminum alloy sheet, the sheet was launched downwards. When the discharge energy reached a certain value, the sidewall and bottom area of the sheet would be impacted at high speed by the die. Meanwhile, high-speed impaction electromagnetic deformation using a cylindrical die was shown in Figure 1b. The cylindrical die cavity is a closed-round die, which has a diameter of 100 mm and an entry radius of 10 mm. The 180 mm diameter workpiece was placed below the coil. All high-speed impaction electromagnetic deformation experiments were conducted in the same manner. All the specimens formed in this study were printed on circular grids with a diameter of 2.5 mm. After the electromagnetic impaction deformation experiment, a circular grid would have an ellipse shape. A digital camera was used to capture the images of the selected grids. The corresponding major and minor strains were calculated from the deformed grids using an automatic grid analyzer software. 

## 3. Numerical Simulation

### 3.1. Magnetic Field Model

According to Maxwell’s equation, the magnetic field equations are as follows:

(1) $$\nabla \times \vec{E}=-\frac{\partial \vec{B}}{\partial t}$$

(2) $$\nabla •\vec{B}=0$$

(3) $$\nabla •\vec{H}=\vec{J}$$

(4) $$\vec{B}=\mu \vec{H}$$

(5) $$\vec{J}=\sigma \vec{E}$$

where $\vec{E}$ is the electric intensity (V/m), $\vec{B}$ is the magnetic flux density (T), $\vec{H}$ is the magnetic intensity (A/m), $\vec{J}$ is the current density (A/m^2^), μ is the permeability (H/m), and σ is the conductivity (m/s). 

The magnetic vector potential usually chooses $\vec{A}$ as a system variable in the analysis of the magnetic field such that

(6) $$\vec{B}=\nabla \times \vec{Α}$$

(7) $$\vec{E}=-\frac{\partial \vec{A}}{\partial t}$$

Thus, the equation obtained is

(8) $$\frac{1}{\mu }\nabla \times (\nabla \times \vec{A})=-\sigma \frac{\partial \vec{A}}{\partial t}$$

where $-\sigma \frac{\partial \vec{A}}{\partial t}$ is the induced current density in a certain area of the workpiece. 

The magnetic force density is expressed as follows:

(9) $$\vec{F}=\vec{J}\times \vec{B}=\frac{1}{\mu }(\nabla \times \vec{B})\times \nabla \times \vec{A}=\frac{1}{\mu }\left[\nabla \times (\nabla \times \vec{A})\right]\times \nabla \times \vec{A}$$

Hence, the magnetic forces can be expressed as $\vec{A}$. The magnetic forces from the magnetic model can be obtained and then be taken as the load input to the constitutive model.

### 3.2. Constitutive Model

**Figure 2:**
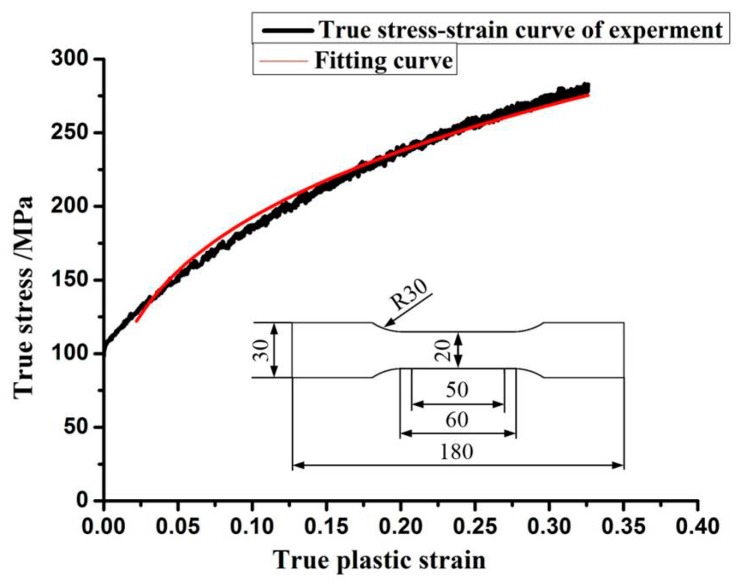
The experimental true stress-strain curve of 5052 aluminum alloy and the fitting curve.

To predict the high-speed electromagnetic deformation process, an appropriate constitutive model was selected based on material deformation behavior at high strain rates. Temperature can be ignored during the electromagnetic forming process because time is extremely short while forming [12,13]. The material behavior will change due to the strain rate effect. To consider the effect of high strain rate on the deformation process, some scholars [14,15] have used viscoplastic material behavior with rate-dependence law, that is, the Cowper-Symonds (C-S) constitutive model, to analyze the electromagnetic forming process. A good agreement is obtained between predicted and the experimental data. Thus, the C-S constitutive model was also used to predict the high strain-rate forming process in the current study. This model is given as

(10) $$\sigma =\sigma_{y}\left[1+(\frac{\overset{•}{\varepsilon }}{p}{)}^{m}\right]$$

where σ is the dynamic flow stress, $\sigma_{y}$ is the quasi-static constitutive behavior of the sheet (Equation (11)), $\overset{•}{\varepsilon }$ is the plastic strain rate, and *p* and *m* are the C-S strain rate parameters. For aluminum alloy, *p* = 6500 s^−1^ and *m* = 0.25 are the specific parameters according to Liu et al. [16,17]. The Equation (11) is exponential model that can accurately describe a large plastic deformation for most metal materials (such as aluminum alloy, magnesium alloy, ferroalloy, and copper). The characteristics of strain hardening and the large plastic deformation during quasi-static forming can be expressed by this model. The elastic modulus (*E*) is assumed to be constant for the elastoplastic stage. The material volume is assumed to be incompressible during the plastic deformation, the poisson ratio is considered as a constant. By fitting the quasi-static constitutive model (Equation (11)), the model parameters *k* = 376.8 MPa and *n* = 0.3 can be obtained. The *k* and *n* are the strain hardening coefficient and hardening exponent, respectively. The fitted curve of the true stress vs. strain is highly consistent with the experimental data, when the plastic strain is ≥0.02 (Figure 2). The electromagnetic deformation is a large plastic deformation process, the effective plastic strain of 5052 aluminum alloy will reach 0.4. Thus, the parameters of the model can express the behavior of 5052 aluminum alloy (when the plastic strain ≥0.02)

(11) $$\sigma_{y}=k\varepsilon^{n}$$

where $\sigma_{y}$ is the quasi-static stress, and ε is the plastic strain.

### 3.3. Finite Element Modeling of Electromagnetic High-Speed Impaction

**Figure 3:**
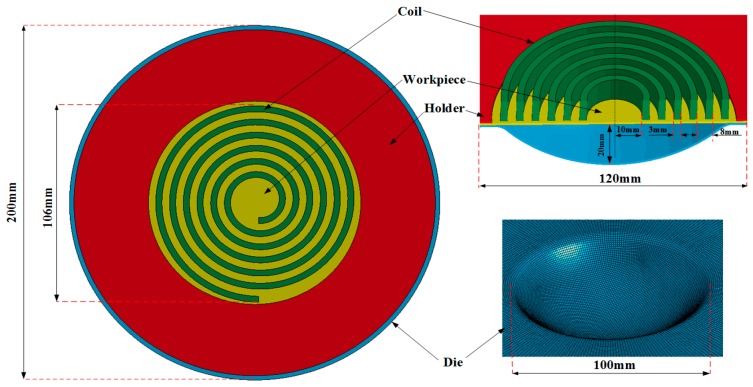
Finite element model of high-speed impaction electromagnetic deformation with a hemispherical die.

**Figure 4:**
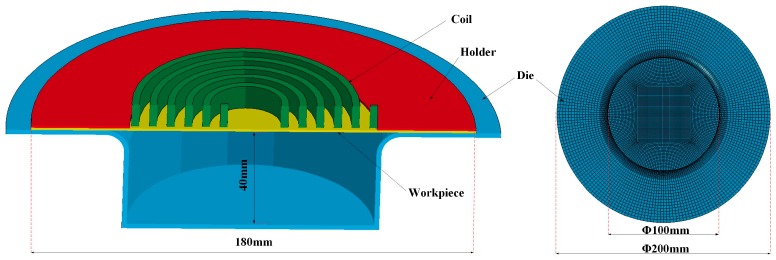
Finite element model of high-speed impaction electromagnetic deformation with a cylindrical die.

**Figure 5:**
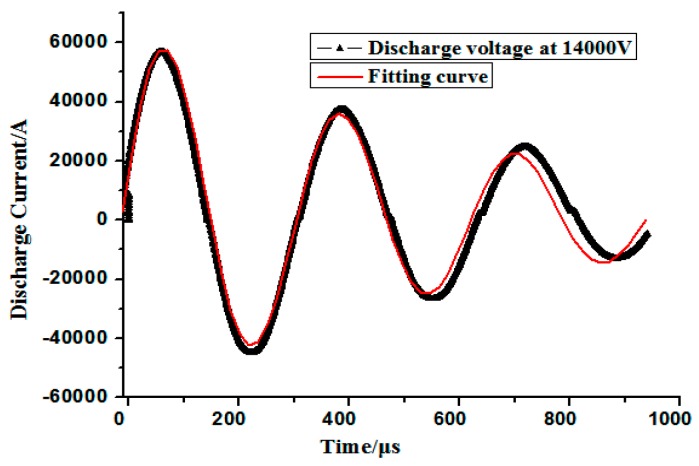
Measured discharge current through the coil in the experiment.

Numerical modeling was performed to investigate the deformation behavior and processes of electromagnetic high-speed impaction. The full coupling of impaction deformation of the electromagnetic field and the structure field was achieved by using finite element software LS-DYNA 9.0. The electromagnetic field in the conductor was analyzed by the finite element method (FEM), whereas the boundary element method (BEM) is used to solve the electromagnetic field of the air. The shell elements of the BEM are generated by the surface of finite elements. The established numerical model consists of a holder, a coil, a workpiece, and a die. All the parts were meshed into eight-node hexahedral elements, and the workpiece was meshed with a higher mesh density to improve the accuracy of the numerical model. The five-layer griddings were distributed along the sheet thickness direction. Coils were meshed with an element size of 0.5 mm in the cross section. The finite element model of high-speed impaction electromagnetic deformation with a hemispherical die was shown in Figure 3. The hemispherical die, coil, and blank holder were set to a rigid body. The holder and the die were considered fixed, and contact conditions were considered between the die and the sheet and between the sheet and the holder. Surface-to-surface contact was defined using the penalty method with a static friction coefficient of 0.2 and a dynamic friction coefficient of 0.1 [18]. The finite element model of electromagnetic high speed impaction with a cylindrical die was shown in Figure 4. The boundary conditions and the friction coefficient are the same as those in Figure 3. The coil structure and the geometric size are the same in the two finite element models. Figure 5 shows the discharged current measured in the experiment at a discharge voltage of 14 kV. The experiment data were fitted for the electromagnetic high-speed impaction, and approximately one cycle of the current of roughly 290 μs was inputted as coil excitation in the numerical simulation. The parameters of 5052 aluminum alloys were shown in Table 2. The current loads were applied to the finite element model. The plastic deformation and fracture behavior of the 5052 aluminum alloy sheet on the hemispherical and the cylindrical dies will be predicted.

## 4. Results and Discussion

### 4.1. Forming Limits of 5052 Aluminum Alloy Formed Using Two Different Dies

**Figure 6:**
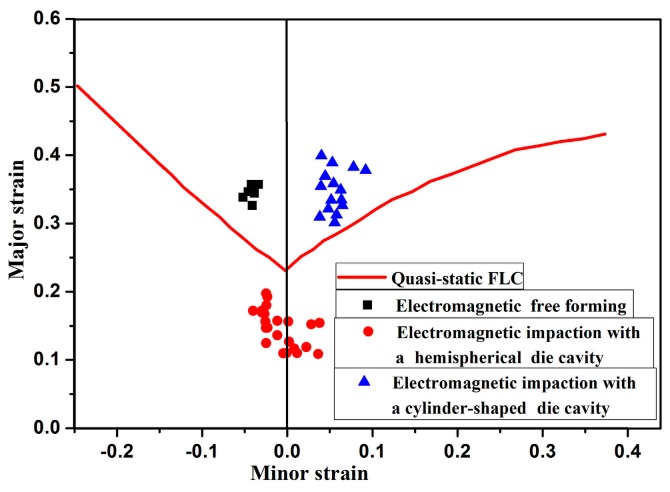
Forming limits of electromagnetic high speed impaction deformation of 5052 aluminum alloy.

Figure 6 shows the formability of 5052 aluminum alloy that was measured experimentally in electromagnetic free forming and electromagnetic impaction deformation with different dies. A formability increase of 24.11% compared with the quasi-static forming conditions was observed in electromagnetic free forming. In high-speed impaction electromagnetic deformation with a cylindrical die, a rebound occurred in the center region of the workpiece, and the strain state was biaxial stretching. A formability increase of 26.67% compared with the quasi-static forming conditions was also observed. However, in electromagnetic impaction with a hemispherical die, a formability decrease of 25% was observed due to sheet-die impaction and serious rebound, which contradicts with the previous reports [20,21,22] that the formability of materials will be increased during high-speed impaction die forming. Therefore, an examination of the forming process in terms of strain rate, impaction pressure stress, and stress state was performed using finite element simulation to provide a reasonable explanation for the unexpected phenomenon that occurred in electromagnetic impaction deformation with a hemispherical die. On this basis, the influence of die geometry on the forming limits of 5052 aluminum alloy in electromagnetic forming is finally discussed.

### 4.2. The Process of High-Speed Impaction Electromagnetic Deformation

**Figure 7:**
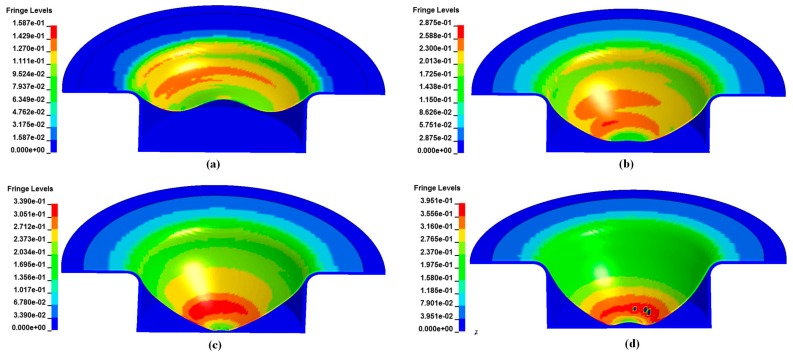
Evolution of the shape and major strain of 5052 aluminum alloy during electromagnetic impaction deformation with the cylindrical die at different times. (**a**) 80 μs; (**b**) 160 μs; (**c**) 200 μs; (**d**) 300 μs.

The evolution of the shape and major strain of the alloy sheet in electromagnetic impaction deformation with the cylindrical die is shown in Figure 7. The sidewall region near the half radius of the sheet was first deformed. Then, deformation at the center, followed by sheet-die impaction at the bottom occurred. Finally, a rebound was observed at the bottom. Consequently, the maximum plastic strain was below the apex region and a crack appeared immediately below the impaction region. 

**Figure 8:**
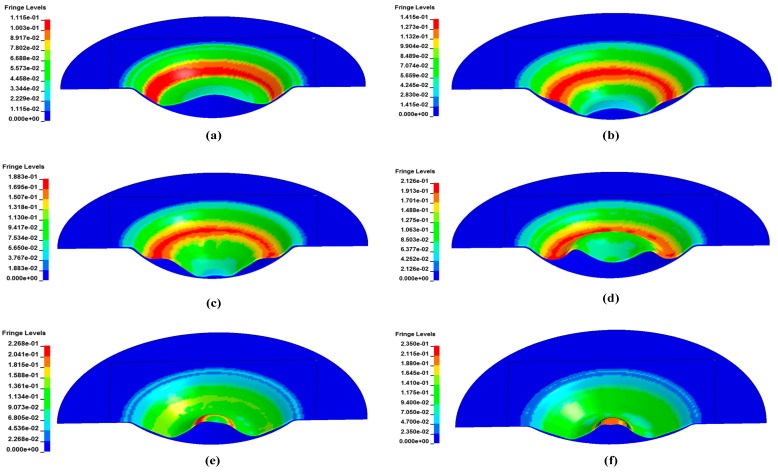
Evolution of the shape and major strain of 5052 aluminum alloy during high-speed impaction electromagnetic deformation with the hemispherical die at different times. (**a**) 80 μs; (**b**) 120 μs; (**c**) 160 μs; (**d**) 240 μs; (**e**) 300 μs; (**f**) 340 μs.

Figure 8 shows the shape variation and major strain via numerical simulation of electromagnetic impaction with the hemispherical die. In the initial deformation stage, the specimen periphery region impacted with the sidewall of the hemispherical die under the driving force of electromagnetic repulsion. Given the restraint of the hemispherical die, the specimen periphery region was extruded upward when the center region of the specimen moved at a high speed toward the bottom of the die (Figure 8c). When the center region of the specimen impacted with the bottom of the die, the center region suffered a serious rebound. Under the action of the electromagnetic force, the sidewall region of the sheet gradually moved downward again (Figure 8d). The center region of the specimen changed from concave downward to upward convex when it impacted with the bottom of the die, and the specimen center region changed from extruding to stretching. Finally, a crack appeared in the apex region of the specimen under high-speed rebound.

### 4.3. Strain Rate

**Figure 9:**
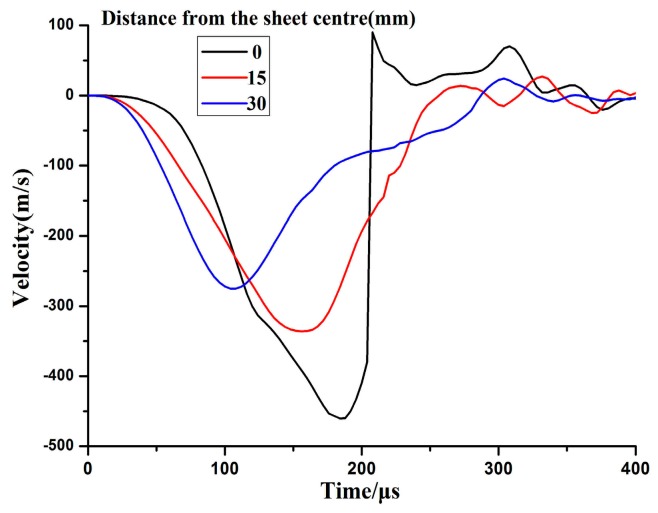
Velocity variation of electromagnetic impaction with the cylindrical die.

**Figure 10:**
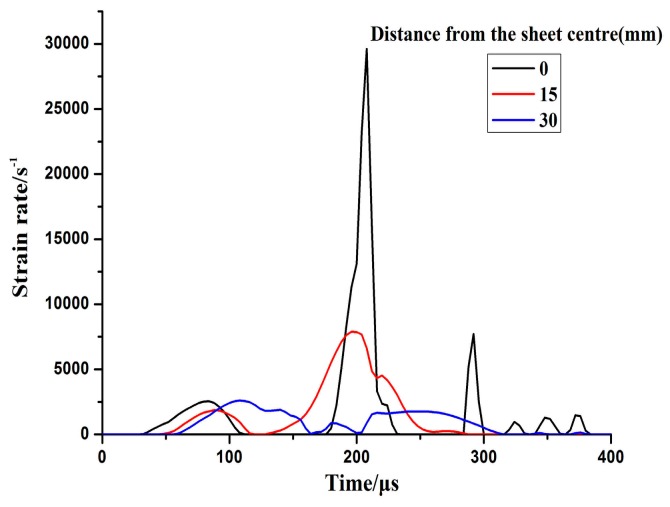
Evolution of strain rate while impacting with the cylindrical die.

Figure 9 shows the velocity variation of electromagnetic impaction deformation with the cylindrical die. The downward maximum velocity of the center point reached 460 m/s. The sheet impacted with the bottom of the cylindrical die at high speed, and a serious upward rebound occurred at the center. However, the upward velocity of the center region increased slightly at close to 290 μs because the rebounding speed continued to increase under the action of inertia. A remarkable increase occurred in the strain rate when the sheet impacted with the cylindrical die as shown in Figure 10. The maximum strain rate was approximately 30,000 s^−1^ when the sheet impacted with the bottom of the cylindrical die at high speed.

**Figure 11:**
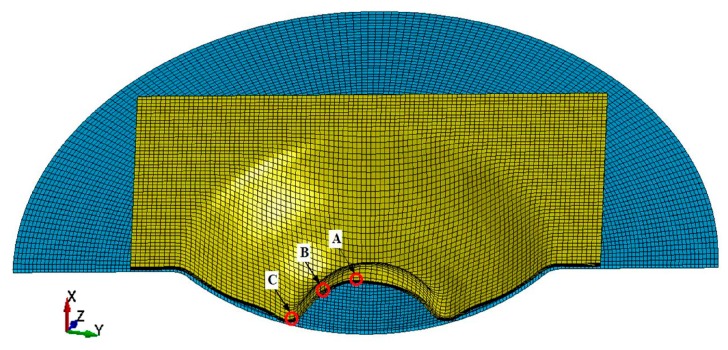
Locations of interest in the specimen that impacted hemispherical die along the radial direction.

**Figure 12:**
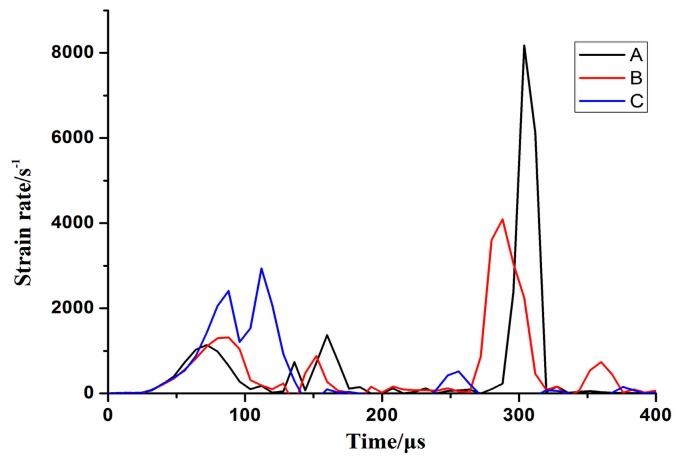
Evolution of strain rate when the sheet impacted with the hemispherical die.

Figure 11 shows the several positions of interest located along the radial direction of the sheet that impacted with the hemispherical die. Element A was located at the center of the sheet, element B was 15 mm away from the center, and element C was 30 mm away from the center. Figure 12 shows the evolution of the effective strain rate when the sheet impacted with the hemispherical die. The maximum effective strain rate was less than 3000 s^−1^ when the sheet impacted with the sidewall and the bottom of the hemispherical die. However, the maximum strain rate of element A was more than 8000 s^−1^ when electromagnetic discharging was completed during the inertia stage. Therefore, the strain rate was different for sheet high-speed impaction with different die geometries at the same discharge energy. The value of strain rate with the cylindrical die was almost four times higher than that with the hemispherical die.

### 4.4. Impaction Pressure Stress

**Figure 13:**
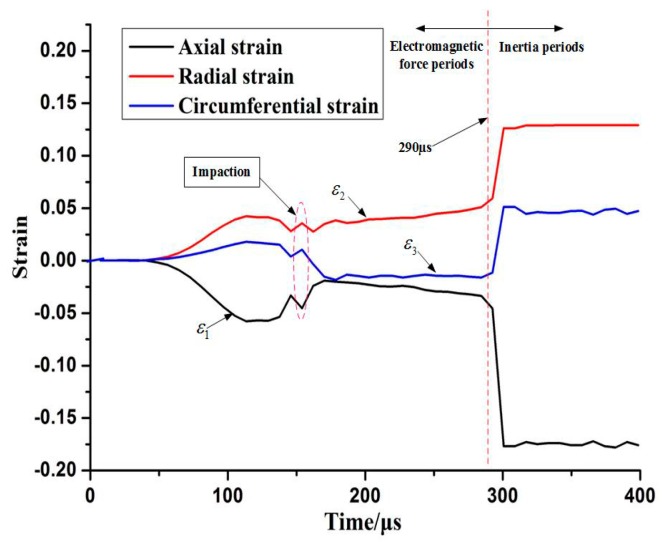
Strain variation of center Element A with time while impacting with the hemispherical die.

**Figure 14:**
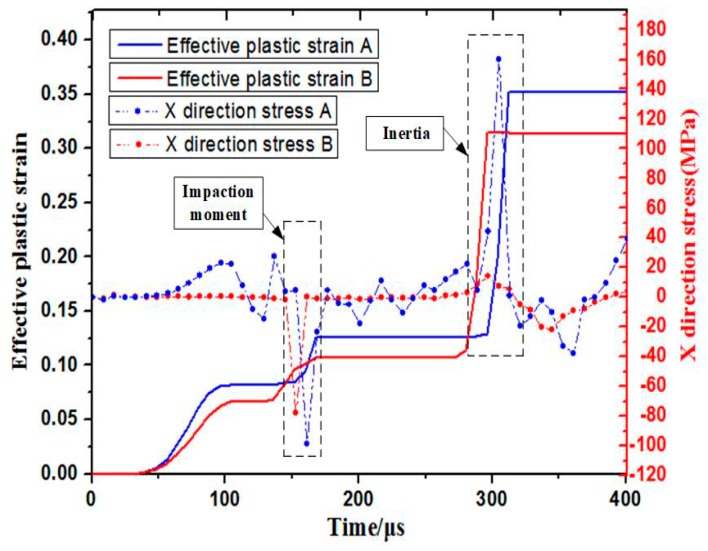
Effective plastic strain and axial stress distribution over time.

Figure 13 shows the time–strain curve of the center region when it impacted with the hemispherical die. The $\varepsilon_{1}$, $\varepsilon_{2}$ and $\varepsilon_{3}$ denote the axial strain, radial strain, and circumferential strain of element A, respectively. Plastic strain increased slightly when the center region of the sheet impacted with the bottom of the hemispherical die at high speed at a time of 160 μs. The circumferential strain of element A changed from positive to negative after the impact. This result indicated that the center region suffered from circumferential extrusion via the sidewall deformation. The squeezing effect was gradually enhanced, particularly by the upward rebound in the center region. However, when electromagnetic discharging was completed during the inertial periods, the absolute value of axial strain increased significantly after 290 μs compared with the impaction process. Therefore, plastic deformation mainly occurred during the inertial periods after electromagnetic discharging was completed rather than during impaction between the sheet and the hemispherical die. The center region of the sheet was initially subjected to severe circumferential extrusion and then suffered from upward rebound tensile stress under inertial action during the impaction process. Consequently, the combination effect of upward “extrusion-stretching” resulted in the fracture of the sheet’s center region. Figure 14 shows the effective plastic strain and axial stress distribution of element A and B over time. When the sheet impacted with the surface of the hemispherical die at high speed, the effective plastic strain did not increase significantly. By contrast, the plastic strain increased rapidly when electromagnetic discharging completed. Element A was subjected to the interaction between tensile stress and compressive stress before impaction. When element A of the sheet impacted with the bottom of the hemispherical die at high speed, the center of the sheet was subjected to impaction stress (approximately 100 MPa). However, when electromagnetic discharging was completed, the sheet’s center region suffered from considerable tensile stress due to the inertia effect.

**Figure 15:**
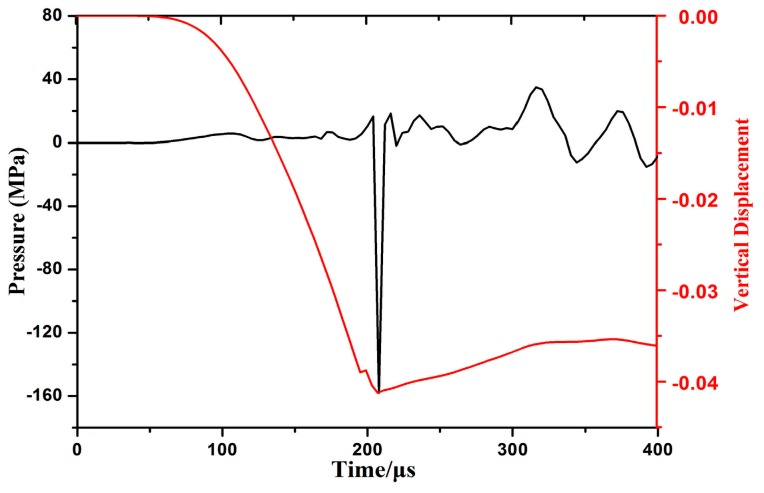
Displacement and pressure of the center region of the sheet while impacting with the cylindrical die.

Figure 15 shows pressure stress and displacement as a function of time for the center of the specimen while impacting with the cylindrical die. Axial pressure at the center was almost zero before 200 μs and then suddenly increased dramatically to approximately 160 MPa at 210 μs as center region impacted with the cylindrical die at high speed. Pressure occurred during shaking up and down under inertial action. The displacement of the center reached the maximum value at the impaction moment, and then a rebound occurred in the center region and displacement decreased. The center region of the sheet nearly reached maximum deformation speed when it impacted with the die. The center region of the sheet impacted with the die bottom as plastic flow increased during high-speed impaction because sufficient plastic deformation nearly occurred in the sheet. 

### 4.5. Stress State

**Figure 16:**
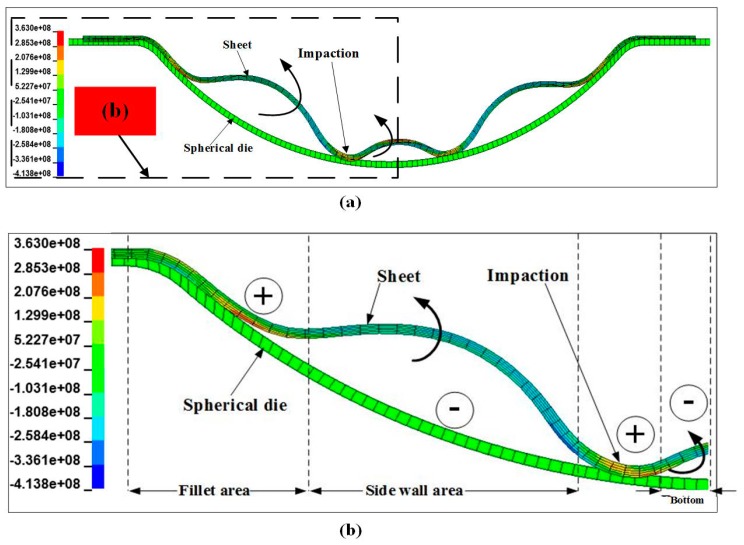
Contours of radial stress distribution of the sheet when it impacted with the hemispherical die. (**a**) entire contour; (**b**) half contour relative to (**a**).

The radial stress state of the sheet is highly complicated during a continuous impaction process. Figure 16a shows the entire contour distribution of the radial stress when the sheet impacted with the hemispherical die. To analyze the stress state in detail, half of the contour is shown in Figure 16b. The sheet was divided into four sections, namely, the fillet region, sidewall region, impaction region, and bottom. The fillet region suffered from tensile stress, whereas the sidewall region of the specimen mainly suffered from compressive stress. An upward extrusion deformation occurred at the sidewall area, and the impaction region suffered from tensile stress at the radial direction. However, the bottom of the specimen was subjected to serious radial compression (over 300 MPa) because further downward deformation was restricted. The improvement of plastic deformation will be inhibited. 

**Figure 17:**
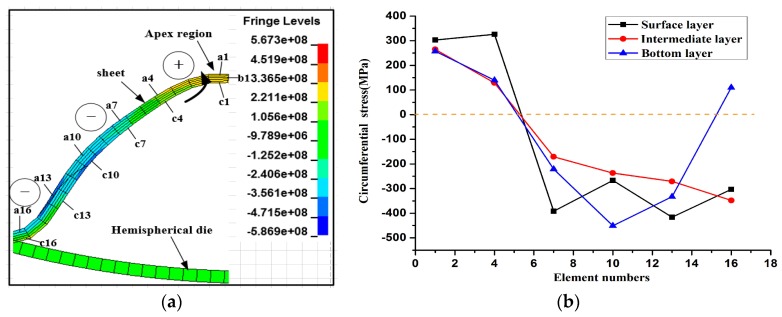
Distribution of the circumferential stress before fracture occurs in the sheet’s center region. (**a**) Contours of the circumferential stress of sheet’s center impaction rebound (**b**) Circumferential stress distribution of the elements in different layers.

Figure 17 shows the contours of the circumferential stress before the sheet’s center region was fractured. The specific elements within the impaction rebound zone were analyzed. The sheet was divided into five layers in the thickness direction. To better analyze the stress state, the surface, intermediate, and bottom layers were selected for analysis. The labels a, b, and c represent the surface, intermediate, and bottom layers, respectively. The element numbers were 1, 4, 7, 10, 13, and 16 from sheet’s center to the sidewall. The circumferential stress was basically negative along the radius direction except for the sheet’s center region, thereby indicating that the sheet’s sidewall suffered from compression throughout the circumferential direction, and only the apex region elements of the sheet exhibited bidirectional tensile stress. Figure 17b shows the circumferential stress distribution of the elements in the different layer illustrated in Figure 17a. The stress state of the same radius at different layers was nearly identical except for element c16. The surface layer element a16 and intermediate layer element b16 were under compression stress, and the bottom layer suffered from tensile stress for element c16 due to upward bending after impacting with the die. Elements 1 to 4 of the sheet’s center region suffered from the tensile stress at all the layers. By contrast, elements of the other sidewall region were mainly subjected to extrusion stress at the circumferential direction. Consequently, the apex region suffered from tensile stress and simultaneously, the sidewall region was subjected to large inward extrusion stress, thereby causing fracture in the center region at an early stage.

### 4.6. Discussion on the Influence of Die Geometry on Formability

The high-speed impaction formability observed in the specimen was dependent on die geometry, which is related to strain rate, pressure stress, and stress state. The strain rate of impacting with the cylindrical die increased considerably compared with the hemispherical die. The strain rate reached nearly 30,000 s^−1^, and the major strain’s relative improvement was approximately 26.67% in formability. Consequently, in the electromagnetic high-speed impact with the hemispherical die, the strain rate did not achieve a higher value compared with impact with the cylindrical die due to die restriction. Moreover, the maximum strain rate was only approximately 1700 s^−1^ when the sheet impacted with the bottom of the hemispherical die. In the electromagnetic high-speed impaction with the hemispherical die, the maximum strain rate was recorded when electromagnetic discharging was completed, which differed from the time that the sheet’s center region impacted with the hemispherical die. 

Axial pressure stress underwent a “whipping” and increased formability when the sheet impacted with the bottom of the cylindrical die. Axial impaction pressure suddenly increased dramatically (approximately 160 MPa), which exceeded the sheet’s yield strength when the sheet impacted with the cylindrical die at high speed. A high impaction pressure can promote the plastic flow of the material. Therefore, the formability of the sheet can be improved to some extent. However, when the sheet impacted with the hemispherical die, the sheet’s center was subjected to impaction pressure stress of approximately 100 MPa, and impaction compressive stress did not reach the sheet’s yield strength (101.4 MPa). Plastic deformation did not evidently occur during the impaction process. Thus, this condition may be one of the reasons why the sheet’s electromagnetic impaction with the hemispherical die did not increase formability. 

The deformation of sheet’s peripheral and center regions was severely constrained by the die when the sheet impacted with the hemispherical die at high speed. The sheet’s bottom region was subjected to radial compressive stress at least thrice the axial impaction pressure stress, which resulted in the radial compression state during impaction. Moreover, when the center region of the sheet impacted and rebounded upward, the apex region suffered from tensile stress while the sidewall region was simultaneously subjected to increased circumferential extrusion stress, which accelerated the onset of fracture in the center region. Consequently, the combination effect of upward “extrusion-stretching” led to the premature rupture of the sheet’s center region, which also resulted in formability decrease in the electromagnetic impact with the hemispherical die.

### 4.7. Verification

**Figure 18:**
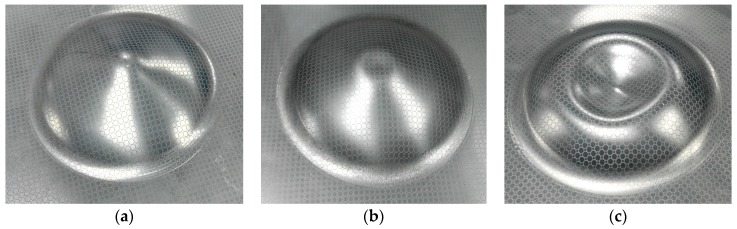
Formed specimens of electromagnetic impaction deformation with the hemispherical die under different discharge energy values: (**a**) 6.8 kJ, (**b**) 8.6 kJ, and (**c**) 15.3 kJ.

Figure 18 shows the deformed specimens obtained under electromagnetic impaction deformation with the hemispherical die at different discharge energy values. The discharge energy that formed the specimen in Figure 18a was 6.8 kJ (corresponding to 8.0 kV) which caused the sheet to fill in the die cavity without impaction. The discharge energy was increased to 8.6 kJ (corresponding to 9.0 kV) such that the specimen impacted with the die at the apex region, and the center region of the specimen was flattened to replace the tip, as shown in Figure 18b. Figure 18c shows that the center region of the specimen experienced a severe rebound under the reacting force of the die when the sheet impacted at high speed with the bottom of the hemispherical die at a discharge energy of 15.3 kJ (corresponding to 12 kV). 

**Figure 19:**
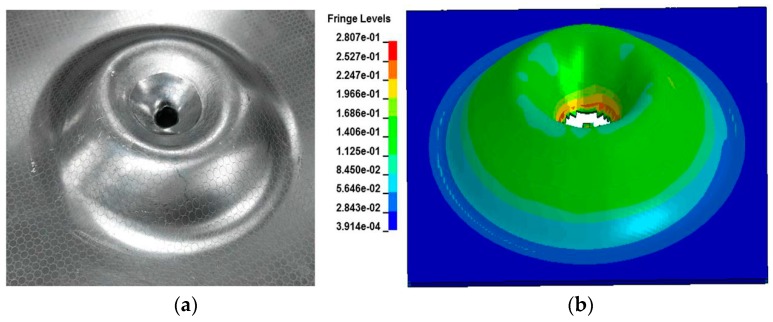
Experimental and predicted fracture specimen impaction with the hemispherical die under a discharge voltage of 14 kV: (**a**) Experiment and (**b**) Simulation result.

Figure 19a shows a severe circumferential crack at the apex of the specimen when the discharge energy reached 20.9 kJ (corresponding to 14 kV). Figure 19a also shows the extremely rough sidewall region of the specimen surface that experienced serious impaction with the hemispherical die. The fracture location appeared at the apex of the rebound region. The simulation results (Figure 19b) predicted a highly similar circumferential crack near the apex of the specimen, which indicated that the maximum equivalent plastic strain in the apex region and the maximum damage also occurred in the same region. Consequently, the elements removal happened in this region. 

**Figure 20:**
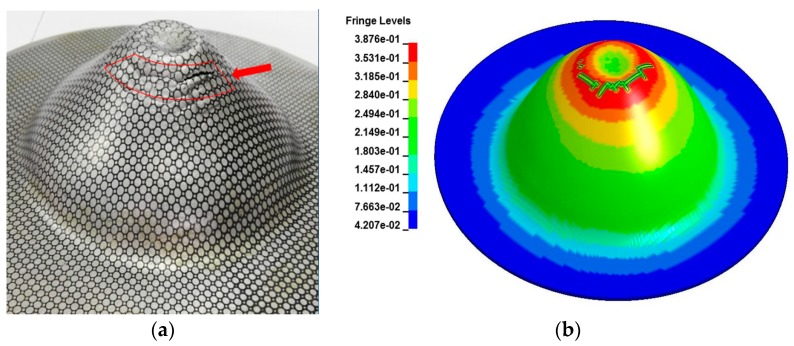
Experimental and predicted fracture specimen impaction with the cylindrical die under a discharge voltage of 14 kV: (**a**) Experiment and (**b**) Simulation result.

Figure 20 shows the experimental and predicted damage specimen impaction with the cylindrical die. The center of the specimen experienced serious impaction with the cylindrical die cavity. The specimen produced serious necking and cracking below the top of the collision region, which was not initiated at the apex of the specimen rebound region. The failure mode of the specimen differed from that of the electromagnetic impaction with the hemispherical die. 

## 5. Conclusions

In the forming of a hemispherical-shaped part, an interesting phenomenon was observed that the high-speed impaction was not always beneficial for the increase of forming limit, and the die geometry could affect the formability of 5052 aluminum alloy sheet with sheet high-speed impaction. This article focusses on reporting the occurrence and the explanation of this phenomenon. The following conclusions could be drawn:

(1) The influence of boundary conditions on formability was investigated in sheet high-speed electromagnetic impaction deformation. When the sheet impacted with the cylindrical die cavity, the major strain of 5052 aluminum alloy improved by approximately 26.67% compared with that of the conventional forming limit. However, when the sheet impacted at high speed with the hemispherical die, the formability of 5052 aluminum alloy was not increased significantly, but instead, decreased by approximately 25% compared with that of the conventional forming limit. Therefore, the high-speed impaction electromagnetic deformation may not improve formability when the deformation of workpiece’s sidewall and bottom was severely constrained by die geometry. Our findings will provide a reference in electromagnetic forming in the future to manufacture ideal hemispherical parts.
(2) The maximum effective strain rate was approximately 30,000 s^−1^ when the sheet impacted to the cylindrical die at high speed. However, this rate was only 1700 s^−1^ when the specimen impacted to the hemispherical die at the same energy level. The plastic flow of sheet’s center region was inhibited, and the strain state changed from biaxial stretching to plane strain. The fracture or necking locations of the sheet evidently differed between the hemispherical die and cylindrical die in electromagnetic impaction deformation.
(3) The impaction pressure was strongly dependent on the discharge energy and the die geometry in sheet electromagnetic impaction deformation. The stress state effect of “extrusion-stretching” resulted in the fracture of the sheet’s center region, which caused the formability decrease with the hemispherical die. In order to improve formability in the high-speed impaction electromagnetic deformation, it was necessary to get the higher strain rate and impaction pressure between the sheet and the die.

## Figures and Tables

**Table 1:** Chemical composition of the 5052 aluminum alloy sheet.

Chemical Element	Si	Fe	Cu	Mn	Mg	Cr	Zn	Al
*Mass fraction (%)*	0.06	0.29	0.01	0.06	2.5	0.16	0.01	Balanced

**Table 2:** The forming coil parameters, material parameters of 5052 aluminum alloy sheet.

***Forming Coil (Copper) Parameters***				***5052 Aluminum Alloys Parameters***								
***Relative Permeability***		***Resistivity (Ω·m)***	***Inductance (H)***	***Relative Permeability***		***Resistivity (Ω·m)***	***ρ (kg/m^3^)***		***E (GPa)***		***Poisson’s Ratio***	
1		1.72 × 10^−8^	1.12 × 10^−5^	1		4.93 × 10^−8^	2700		72		0.3	
***C-S constitutive model parameters***				***GTN damage model parameters* [6,19]**								
***K***	***n***	***p***	***m***	***f_0_***	***f_c_***	***f_F_***	***f_N_***	***S_N_***	$\varepsilon_{N}$	***q_1_***	***q_2_***	***q_3_***
*376.8*	*0.3*	*6500*	*0.25*	0.002918	0.030103	0.04854	0.0249	0.1	0.3	1.5	1	2.25
